# Survivability of *Vibrio cholerae* O1 in Cooked Rice, Coffee, and Tea

**DOI:** 10.1155/2013/581648

**Published:** 2013-07-25

**Authors:** John Yew Huat Tang, Bariah Ibrahim Izenty, Ahmad Juanda Nur' Izzati, Siti Rahmah Masran, Chew Chieng Yeo, Arshad Roslan, Che Abdullah Abu Bakar

**Affiliations:** ^1^Faculty of Food Technology, Universiti Sultan Zainal Abidin, Gong Badak Campus, 21300 Kuala Terengganu, Terengganu, Malaysia; ^2^Faculty of Agriculture and Biotechnology, Universiti Sultan Zainal Abidin, Gong Badak Campus, 21300 Kuala Terengganu, Terengganu, Malaysia

## Abstract

This study aimed to investigate the survival of *Vibrio cholerae* O1 in 3 types of preparation for cooked rice, *Oryza sativa* L., (plain rice, rice with coconut milk, and rice with ginger); coffee, *Coffea canephora*, (plain coffee, coffee with sugar, and coffee with sweetened condensed milk); and tea, *Camellia sinensis*, (plain tea, tea with sugar, and tea with sweetened condensed milk) held at room temperature (27°C). The survival of *V. cholerae* O1 was determined by spread plate method on TCBS agar. Initial cultures of 8.00 log CFU/mL were inoculated into each food sample. After 6 h incubation, significant growth was only detected in rice with coconut milk (9.67 log CFU/mL; *P* < 0.05). However, all 3 types of rice preparation showed significant growth of *V. cholerae* after 24 h (*P* < 0.05). For coffee and tea preparations, *V. cholerae* survived up to 6 h in tea with condensed milk (4.72 log CFU/mL) but not in similar preparation of coffee. This study showed evidence for the survivability of *V. cholerae* in rice, coffee, and tea. Thus, holding these food and beverages for an extended period of time at room temperature should be avoided.

## 1. Introduction

The World Health Organization [1] reported the reemergence of cholera as a major infectious disease which posed a global threat to public health, especially in developing countries. In 2009, cholera recorded a fatality rate of 2.24% from a total of 221,226 reported cases [2]. Sporadic outbreaks of cholera can happen in any areas with inadequate supply of water, sanitation, food safety, and hygiene practices [3]. Outbreaks of cholera have been reported since the dawn of ancient civilization in the Ganges delta, India [4].

Although *Vibrio cholerae *is the causal agent for cholera, it is noteworthy that only a small number of *V. cholerae* are capable of producing cholera toxin which causes the clinical symptom [5] presented as acute diarrhea, commonly known as “rice water” stool. 

Water is recognised as the main mode of cholera transmission in areas where clean and adequate water supply is limited. In countries where potable water is available, food would be the main vehicle for cholera outbreaks [6]. *V. cholerae *have been reported to cause several outbreaks which implicated food contaminated with *V. cholerae* Ol [7], but person-to-person transmission is uncommon [7]. Outbreaks have been reported to be associated with the consumption of raw or undercooked seafood, and this has been recognized to be a very important factor contributing to foodborne cholera [8]. However, other food such as contaminated cooked rice and fresh produce had also been reported [8, 9]. Ready-to-eat (RTE) foods can be contaminated with *Vibrio* following poor hygiene practices by food handlers [10–12], and the organism can multiply rapidly at ambient temperatures.

Rice is a staple food in the Southeast Asian countries and is thus a main source for *V. cholerae *contamination. There is currently very little known regarding the survivability of *V. cholerae *in various types of cooked rice and beverages that are commonly found in Malaysia. Our previous study [13] had reported on the survival pattern of *V. cholerae* O1 on three types of rice kept in an opened container, and the current study aims to investigate the survivability of *V. cholerae *O1 in different preparations of cooked rice, as well as the common beverages tea and coffee, along with other ingredients in an enclosed container. This study will provide useful information with regards to *V. cholerae *behaviour in food and beverages that are common in Malaysia. As such, the intervention and proper practices can be determined to reduce the risk of cholera outbreak by food handlers, especially street foods. 

## 2. Materials and Methods

### 2.1. *Vibrio cholerae*

*V. cholerae *O1 serotype Inaba was obtained from Kyoto University, Japan. The bacterium was kept in glycerol stock (−20°C) and was used throughout this study.

#### 2.1.1. Preparation of *V. cholerae *for Experiments

*V. cholerae *was prepared as described in our previous study [13]. *V. cholerae *O1 from a stock culture was streaked onto TCBS agar and incubated at 37°C for 24 h. Isolated *V. cholerae* colony was inoculated into alkaline peptone water and incubated in shaker incubator (150 rpm) (Infors HT Ecotron, Basel, Switzerland) at 37°C for 22 h. The culture was centrifuged, and the bacterial pellet was resuspended in phosphate-buffered saline (PBS). Absorbance of the bacterial suspension at 625-nm wavelength was adjusted to a reading of 1.89, which corresponded to about 5 × 10^9^ CFU/mL.

#### 2.1.2. Enumeration of Spiked *V. cholerae*

The spread plate method was performed as described in the previous study [13]. Each dilution was plated in triplicates, and the plates were incubated at 37°C for 24 h. Yellow colonies on replicate plates were counted and expressed as mean *V. cholerae *CFU per (g/mL).

#### 2.1.3. Preparation of Cooked Rice for Experiments

Nonglutinous white rice (*Oryza sativa *L.) was used throughout this study. Plain rice was prepared using 5 g of white rice washed twice with 10 mL of sterile distilled water. The washed rice was transferred to a large test tube, then 8 mL of sterile distilled water was added, and the mixture was cooked in a beaker with boiling water. The cooked rice with coconut milk was prepared using 5 mL of distilled water together with 3 mL of coconut milk while the cooked rice with ginger used 8 mL of distilled water together with 0.1 g of ginger. The time taken to cook the rice in boiling water was about 15 min for the white rice. All cooked rice preparations were left in an incubator (Infors HT Ecotron) to cool down to 27°C. A new batch was freshly prepared on the day of each experiment.

#### 2.1.4. Preparation of Coffee for Experiments

Instant coffee powder (*Coffea canephora*) was used throughout this study. Plain coffee was prepared by adding 1 g of instant coffee powder into 100 mL of boiled distilled water. Coffee with sucrose (hereafter referred to as sugar) was prepared using 1 g of instant coffee powder with 7 g of sugar in 100 mL of boiled distilled water, while coffee with condensed milk was prepared using 1 g of instant coffee powder with 15 g of condensed milk in 100 mL of boiled distilled water. All the coffee preparations were left in the incubator (Infors HT Ecotron) to cool down to 27°C. A new batch was freshly prepared on the day of each experiment.

#### 2.1.5. Preparation of Tea for Experiments

 Loose black tea (*Camelia sinensis*) was used throughout this study. Plain tea was prepared by adding 1 g of loose black tea to 100 mL of boiled distilled water. Tea with sugar was prepared using 1 g of loose black tea together with 5 g of sugar in 100 mL of boiled distilled water, while tea with condensed milk was prepared using 1 g of loose black tea together with 15 g of condensed milk in 100 mL of boiled distilled water. All the tea preparations were left in the incubator (Infors HT Ecotron) to cool down to 27°C. A new batch was freshly prepared on the day of each experiment. 

#### 2.1.6. Survival of *V. cholerae *Inoculated onto Cooked Rice

Three grams of the cooked rice was transferred to each of the five universal bottles [13]. An estimated 1 × 10^8^ CFU of *V. cholerae *in 20 *μ*L of PBS was dispensed randomly as small droplets on each of the rice clumps for incubation time of 0, 1, 3, 6, and 24 h at 27°C in the incubator (Infors HT Ecotron). The bottles were loosely capped. Immediately after the last sample was inoculated (0 h exposure), 5 mL of PBS was added to the bottle and the rice grains in it were mixed by vortex for 1 min. Determination of viable counts of *V. cholerae *was performed with 100 *μ*L of serial diluted rice suspension on TCBS agar. The same procedure was performed with the remaining inoculated samples after 1, 3, 6, and 24 h of incubation time. *V. cholerae *inoculated into empty bottles were kept under similar conditions to serve as control. Four replicated experiments were performed on each rice preparation. 

#### 2.1.7. Survival of *V. cholerae *Inoculated into Coffee and Tea

Ten millilitres of the prepared coffee (or tea) was transferred to each of the five universal bottles. An estimated 1 × 10^8^ CFU of *V. cholerae *in 20 *μ*L of PBS was inoculated into the coffee (or tea) for incubation time of 0, 1, 3, and 6 h at 27°C in incubator (Infors HT Ecotron). The bottles were loosely capped. Immediately after the last sample was inoculated (0 h exposure), the sample was mixed by vortex for 1 min. Determination of viable counts of *V. cholerae *was performed with 100 *μ*L of serial diluted coffee (or tea) suspension on TCBS agar. The same procedure was performed with the remaining inoculated samples after 1, 3, and 6 h of incubation time. *V. cholerae *inoculated into 10 mL of sterile distilled water was kept under similar conditions and served as a control. Four replicated experiments were performed on each coffee (or tea) preparation.

#### 2.1.8. Determination of pH and a_w_ of Cooked Rice, Coffee, and Tea

The pH and a_w_ of cooked rice, coffee, and tea were taken using pH 211 Microprocessor pH meter (Hanna Instrument, India) and HygroLab 3 Bench (Rotronic Instrument Corp., NY, USA), respectively.

#### 2.1.9. Statistical Analysis

Data collected during the experiment was analyzed using SPSS 17.0 software. The data were analyzed using Kruskal-Wallis one-way analysis of variance and Mann-Whitney *U* test. The significance level was set at *P* < 0.05.

## 3. Results

**Figure 1:**
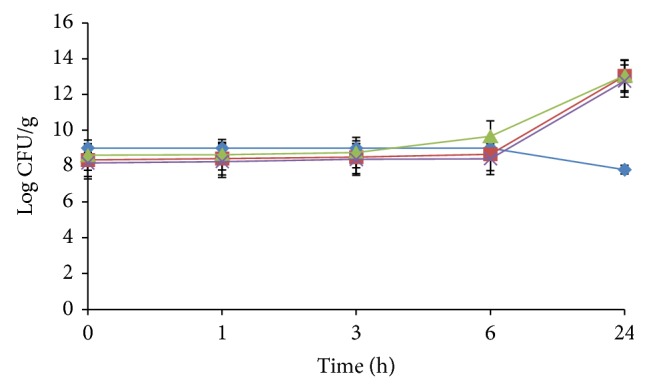
Survival of *V. cholerae* on empty capped bottle as control (♦), clumps of cooked plain rice (■), rice with coconut milk (▲), and rice with ginger (×). Each point is the mean of four replicate experiments.

Figure 1 shows the survival of *V. cholerae *inoculated onto three types of cooked rice. *V. cholerae *survived well in the enclosed empty bottle which was used as the control where its survivability was stable up to 6 h before declining significantly (*P* < 0.05) after 24 h incubation. There was no statistically significant difference up to 6 h incubation for all test samples (control, plain rice, rice with coconut milk, and rice with ginger). There was significant growth (*P* < 0.05) of *V. cholerae *on plain rice, rice with coconut milk, and rice with ginger after 24 h incubation. However, there was no significant difference of *V. cholerae* survivability with regards to the ingredients added. The mean pH recorded for the plain rice, rice with coconut milk, and rice with ginger was 5.34 ± 0.02, 5.63 ± 0.02 and 5.28 ± 0.02, respectively. Mean a_w_ recorded for the plain rice, rice with coconut milk, and rice with ginger were all 0.99 ± 0.00.

**Figure 2:**
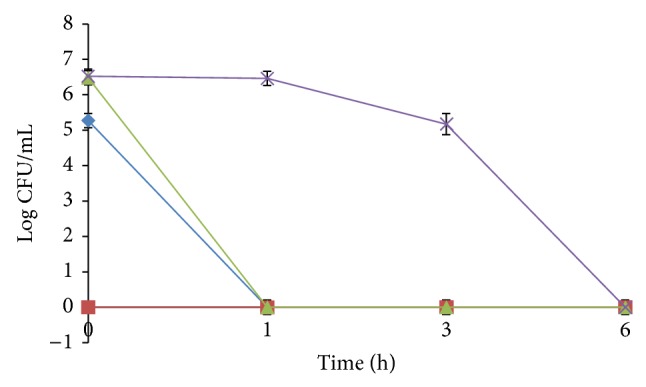
Survival of *V. cholerae* in sterile distilled water as control (♦), plain coffee (■), coffee with sugar (▲), and coffee with condensed milk (×). Each point is the mean of four replicate experiments.

The survival of *V. cholerae *inoculated in three types of coffee preparation is shown in Figure 2. *V. cholerae *survived poorly in the control sterile distilled water with no detectable *V. cholerae *after 1 h incubation. An immediate inactivation of *V. cholerae *was observed in plain coffee with no detection of the organism at 0 h. *V. cholerae *was only detected at 0 h in coffee with sugar while longer survival (up to 3 h) was observed in coffee with condensed milk. There was no *V. cholerae *detected after 6 h incubation in all preparations of coffee. The mean pH recorded for the plain coffee, coffee with sugar, and coffee with condensed milk was 4.68 ± 0.02, 4.81 ± 0.01, and 6.01 ± 0.02, respectively. The mean a_w_ recorded for all three preparations of coffee were 0.99 ± 0.00.

**Figure 3:**
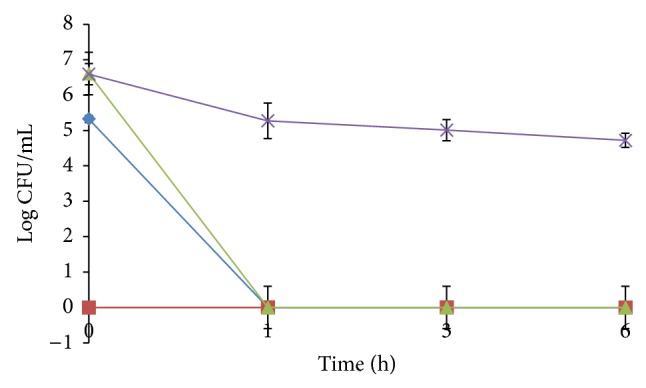
Survival of *V. cholerae* in sterile distilled water as control (♦), plain tea (■), tea with sugar (▲), and tea with condensed milk (×). Each point is the mean of four replicate experiments.

Similar results were obtained for preparations of tea (Figure 3). An immediate inactivation of *V. cholerae *was observed in plain tea with no detection of the organism at 0 h. *V. cholerae *was only detected at 0 h in tea with sugar while longer survival (up to 6 h) was observed in tea with condensed milk. The mean pH recorded for the plain tea, tea with sugar, and tea with condensed milk was 5.18 ± 0.01, 5.29 ± 0.01, and 6.10 ± 0.02, respectively, whereas the mean a_w_ recorded for the three tea preparations were 0.99 ± 0.00.

## 4. Discussion

This study revealed that *V. cholerae *O1 was able to survive in street food and beverages commonly found in Malaysia. Studies have shown that poor food handling had resulted in numerous cases of food contamination with pathogens which eventually resulted in outbreaks [8]. Our previous study had shown that rice was able to support the growth of *V. cholerae *O1 [13]. Several reports also showed the relationship between rice and cholera outbreaks [9, 14–16]. *Nasi lemak* is a popular local food that uses coconut milk and ginger as the ingredients for cooking the rice, and it has been implicated in several cholera outbreaks in Malaysia [6]. The phytoconstituents of ginger have been known for their antibacterial properties. Studies have reported that zingerone and shogaols in ginger have antimicrobial properties against *Staphylococcus aureus, Streptococcus pyogenes, Salmonella enterica* serovar Typhi, and *V. cholerae *[17–19]. However, the amount of ginger that has been added to the rice sample may be too small to show any significant inhibition of *V. cholerae *in the present study. 

In the present study, plain coffee showed instant inactivation of *V. cholerae *O1, and this may be due to the acidic nature of coffee [20]. According to the Public Health Agency of Canada [21], *V. cholerae *can grow very fast under optimum conditions which include pH from 5.00 to 9.60 and water activity of approximately 0.97 to 0.99. Other factors that could inhibit the growth of *V. cholerae *O1 in plain coffee may be the presence of antimicrobial components such as caffeine, volatile and nonvolatile organic acid, phenols, and other aromatic compounds [22–24]. Studies have shown that the coffee extracts were capable of inhibiting bacteria such as *Escherichia coli*, *S. aureus, *and *Streptococcus mutans* [22–24]. Daglia et al. [23] reported that instant coffee possessed high antimicrobial compound in which the acid extract of coffee during roasting process showed significant antibacterial properties. The relative survival of *V. cholerae *O1 was affected by the addition of sugar in the coffee. *V. cholerae *O1 survived up to 1 h in the coffee added with sugar that contains 99.5 to 99.9% pure sucrose. Felsenfeld [25] reported that the presence of sucrose is favourable for the survival of the Vibrios. However, the survival of *V. cholerae *O1 in coffee with sugar did not exceed one hour. In contrast, *V. cholerae *O1 survived up to 3 hours period in the coffee added with condensed milk. An increase in pH to 6.01 of coffee with condensed milk might contribute to the better survival of *V. cholerae *as studies discovered that the near-neutral pH facilitates the survival and multiplication of* V. cholerae* with increasing pH [26]. 

Tea has been well known for its antioxidant and antimicrobial properties [20, 27, 28]. The antimicrobial properties of tea are found in its polyphenol fractions, in particular the compounds epicatechin gallate (ECG) and epigallocatechin gallate (EGCG). *V. cholerae *was not detected after inoculation into the plain tea probably because of the antimicrobial properties of the tea itself [20, 27, 28]. Rabbani and Greenough III [29] discussed the physicochemical characteristics of foods that support the survival and growth of *V. cholerae *O1 which include neutral or an alkaline pH, low temperature, and high-organic content. This was proven in this study as *V. cholerae *was not detectable in the acidic condition (pH 5.18) of the tea samples. Nevertheless, the addition of sugar and condensed milk in tea had improved the survival of *V. cholerae *in the tea as observed in this study. 

As a conclusion, this study clearly showed that *V. cholerae *is able to survive for an extended period of time in rice and coffee or tea that has been prepared with condensed milk, which are popular street food and beverages in Malaysia. Poor hygiene in food and beverage preparations by food handlers may result in cross-contamination that is made worse as the contaminated food (especially rice) is usually held at ambient temperatures for long periods of time, thereby allowing the pathogens to multiply rapidly before the detectable spoilage sign is observed.
